# Effects of different cooking methods of oatmeal on preventing the diet-induced increase of cholesterol level in hypercholesterolemic rats

**DOI:** 10.1186/s12944-015-0138-7

**Published:** 2015-10-24

**Authors:** Yandong Ban, Ju Qiu, Changzhong Ren, Zaigui Li

**Affiliations:** Laboratory of Cereal Science, College of Food Science and Nutritional Engineering, China Agricultural University, Beijing, 100083 China; Institute of Food and Nutrition Development, Ministry of Agriculture, 12 Zhongguancun South Street, Haidian District, Beijing, 100081 China; Oat Engineering Centre, Baicheng Academy of Agricultural Sciences, Baicheng, Jilin 137000 China

**Keywords:** Oatmeal, Brewing, Boiling, Hypercholesterolemia, Rats

## Abstract

**Background:**

The aim of present study is to investigate the influences of brewing and boiling on hypocholesterolemic effect of oatmeal in rats fed with a hypercholesterolemic diet.

**Methods:**

Fifty-six male Sprague–Dawley rats were divided into 5 groups of 8 rats each with similar mean body weights and serum cholesterol concentrations. Rats were fed with the experimental diets containing 10 % of oatmeal from two Chinese oat varieties which were brewed or boiled for 30 days. The lipids levels in serum, liver, and faeces were determined.

**Results:**

The effects of feeding boiled oatmeal on lowering lipid concentrations in plasma and liver were more significant than that of brewed oatmeal (*P* < 0.05). Feeding boiled oatmeal was also more efficient in increasing fecal total lipids, cholesterol and bile acids as compared to feeding brewed oatmeal (*P* < 0.05). Boiled oatmeal had higher apparent viscosity and soluble β-glucan content than the brewed oatmeal did (*P* < 0.05).

**Conclusion:**

These results indicated that the capability of boiled oatmeal in improving cholesterol metabolism is better than that of brewed oatmeal, which is mainly attributed to its higher soluble β-glucan content and apparent viscosity.

## Introduction

Dietary oat effectively lowers the plasma total cholesterol and low-density lipoprotein (LDL)-cholesterol concentrations and, hence, reduces the risk of cardiovascular disease in animal models and hypercholesterolemic subjects [1, 2]. The health benefits of oat are mainly attributed to its high content of nutritional components, including 2.0 ~ 7.5 % of β-glucan [3], 2 ~ 12 % of crude fat [4], 13 ~ 20 % of protein [5] and around 60 % of starch [6]. Previous studies have also shown that dietary full-fat from oat bran could promote excretions of fecal lipids and bile acids. Oat oil has high tocotrienols and sterols contents and, hence, hypocholesterolemic effect. As a result, oat bran with high lipids content lower serum total cholesterol and LDL-cholesterol concentrations [7] as well as the concentrations of rat plasma total cholesterol, LDL-cholesterol the cholesterol, free cholesterol, cholesterol ester and triglyceride levels of liver [8].

According to Wolever et al. [9], dietary oat β-glucan reduces plasma cholesterol total cholesterol and LDL-cholesterol levels by increasing intestinal viscosity, which lowers the reabsorption of bile acids, leading to lower plasma lipids levels. Wood et al. [10] reported that there was a significant correlation between the peak levels of blood glucose concentration and the viscosity of β-glucan. Therefore, it is nutritionally favorable for β-glucan to have a high viscosity. On the other hand, as reported by Tong et al. [11], dietary hull-less barley β-glucan could reduce the concentration of plasma LDL cholesterol in hypercholesterolemic hamsters. However, this hypocholesterolemic effect was weaker than that of oat β-glucan because of the higher viscosity and prebiotic activity of oat β-glucan. Previous study demonstrated that the viscosity and hypocholesterolemic effect of β-glucan was positively correlated with its molecular weight, namely, the molecular weight of β-glucan may influence the hypocholesterolemic effect [12].

Thermal, physical and enzymatic treatments had significant effects on the water solubility and molecular weight of β-glucan and, thereby, could affect the viscosity of hull-less barley [13]. In addition to molecular weight of β-glucan, the soluble β-glucan content can also affect the lipids-lowering effect of oat or hull-less barley because soluble β-glucan can formhighly viscous solutions, which seems to be responsible for reducing the plasma cholesterol in humans and animals [14–16]. In China, brewing and boiling are the most common cooking methods for oatmeal. However, few publications can be found reporting the relationship between the cooking methods and the health impacts of oatmeal, especially the lipid-lowering activity.

The aim of this study was to investigate the effects of oatmeal cooking methods including brewing and boiling on cholesterol metabolism of Sprague–Dawley rats fed with two different varieties of oatmeal. The apparent viscosity, soluble β-glucan and other index of brewed and boiled samples were determined, in order to clarify the differences in their hypocholesterolemic abilities.

## Materials and methods

### Materials

Oatmeals (Avena sativa L., Bayou NO.1 and Bayou NO.8) were provided by Beijing Lipid-lowering Oats Products Development Co. Ltd. Oatmeal Bayou NO.1 contained 12.9 % protein, 75.9 % total starch, 5.6 % crude lipid and 5.6 % crude fiber. Oatmeal Bayou NO.8 contained 16.7 % protein, 68.8 % total starch, 5.5 % crude lipid and 9.0 % crude fiber. The compositions of oatmeals were determined using the methods of AACC-32-23 (2000), AOAC 996.11, GB/T5511-2008, GB/T5512-2008, GB6193-86, and GB/5009.3-2010, respectively.

### Oatmeal treatment

To obtain brewed oatmeal sample, 250 g of oatmeal was mixed with 1 L of 100 °C hot deionized water, and then cooled at room temperature with constant stirring. Boiled oatmeal sample was prepared by cooking 250 g of oatmeal in 1 L of 100 °C hot deionized water with constant boiling for 5 min using an electronic cooker (Povos-PIB07, Shanghai POVOS Electric Works Co. Ltd., Shanghai, China), and then cooling at room temperature.

### Apparent viscosity

The heat treated oatmeal samples were filtrated through a 100 mesh filter cloth, respectively. The apparent viscosity of the filtrate was measured by a digital viscometer (Model NDJ-9S, Shanghai Precision and Scientific Instruments Co., Ltd., Shanghai, China) using a 3^#^ rotor at a shear rate of 3 r/min and 25 °C.

### Soluble β-glucan concentration

The β-glucan concentration in the filtrate of treated oatmeal was determined by an enzymatic method using a mixed-linkage β-glucan assay kit (Megazyme International Ireland, Ltd., Wicklow, Ireland).

### Starch gelatinization

The starch gelatinization was measured using an enzymatic method according to Xiong et al. [17] with some modifications. The treated oatmeal samples were freeze-dried, and then crushed into flours with a cyclone mill. The flour was passed through a 100 mesh sieve. The oatmeal flour (100 mg) was added to 15 mL buffer (0.37 % glacial acetic acid and 0.41 % anhydrous sodium acetate in deionized water) and boiled for 1 h to get a full pasting sample. The full pasting sample was mixed with 1 mL of amyloglucosidase (enzyme activity 100 U/mg) solutions (25 %) and incubated at 40 °C for 1 h in a test tube. The buffer mixed with enzyme solution and without oatmeal flour was used as a blank. After 1 h of incubation, 2 mL of 10 % ZnSO_4_·7H_2_O, 1 mL of 0.5 N NaOH and 7 mL of deionized water were added to the test tube. The reaction solution was filtered by filter paper (Whatman 40^#^) The filtrate (0.1 mL) and copper reagent (2 mL, 40 g NaCO_3_, 7.5 g tartaric acid, and 4.5 g CuSO_4_·5H_2_O in 1 L deionized water) were mixed and boiled for 6 min. Then, the suspension in the tube was mixed with 2 mL molybdic acid solution (70 g molybdic acid, 10 g sodium tungstate, 40 g NaOH, and 250 mL H_3_PO_4_ (85 %) in 1 L deionized water), heated (temperature?) for 2 min, and filled up (with what buffer) to 25 mL. Absorbance at 420 nm of the solutions was measured using a spectrophotometer. Starch gelatinization was calculated using the following formula.

$$ \mathrm{Starch}\ \mathrm{gelatinization}\left(\%\right) = \left({\mathrm{A}}_{\mathrm{test}\ \mathrm{sample}}\hbox{-} {\mathrm{A}}_{\mathrm{blank}}\right)/\left({\mathrm{A}}_{\mathrm{full}\ \mathrm{pasting}\ \mathrm{sample}}\hbox{-} {\mathrm{A}}_{\mathrm{blank}}\right) \times 100\% $$

### Protein dispersibility index

Protein dispersibility index (PDI) was measured using a method described by Iwe et al. [18] with some modifications (). Briefly, the oatmeal flour (15 g) was blended in 250 mL of deionized water for 10 min in a XHF-D HI-speed dispersator (Ningbo Scientific Biotechnology, Ningbo, China) and then centrifuged at 5000 g at 25 °C. The protein concentration of supernatant was determined by Kjeldahl method (AOAC 984.13). The PDI was calculated by the following formula.

$$ \mathrm{P}\mathrm{D}\mathrm{I}\left(\%\right) = \mathrm{Solubility}\ \mathrm{protein}/\mathrm{Total}\ \mathrm{protein}\ \mathrm{content}\ \mathrm{of}\ \mathrm{sample} \times 100\% $$

### Animals and diets

Fifty-six male Sprague–Dawley rats (*n* = 40) were obtained from College of Medicine, Xi’an JiaoTong University (Xi’an, China). All rats were housed individually in stainless steel cages under controlled temperature (22 ± 2 °C), humidity (55 ± 5 %) and air flow conditions with a fixed 12 h light–dark cycle. After 1 week acclimation, rats were divided into 5 groups, namely, Control (C), Brewed Bayou No.1 (Br1), Boiled Bayou No.1 (Bo1), Brewed Bayou No.8 (Br8), and Boiled Bayou No.8 (Bo8). The average body weight of the rats was similar for each group. The rats in different groups were fed with different oatmeals and deionized water for 5 weeks. Experimental diets shown in Table 1 were prepared according to the American Institute of Nutrition (AIN)-93G formula. As shown in Table 1, the cholesterol, bile salts and lard was added to standard diet in order to elevate the concentration of serum cholesterol. Rat feces were collected for 3 days before sacrification. The rats were fasted for 16 h and then sacrificed by the removal of blood from the abdominal aorta. Their livers were quickly dissected and washed in ice-cold saline solution to remove the blood, and then stored at −80 °C in a deep freezer for biochemical analysis.

**Table 1:** Diet composition (g/1000 g diet)

	C	Br1	Bo1	Br8	Bo8
Casein	200	187.1	187.1	183.3	183.3
Corn Starch	397	321.1	321.1	328.2	328.2
Soybean oil	70	64.4	64.4	64.5	64.5
Cellulose	50	44.4	44.4	41.0	41.0
Oatmeal					
Brewed Bayou No.1		100			
Boiled Bayou No.1			100		
Brewed Bayou No.8				100	
Boiled Bayou No.8					100
Maltodextrin 10	132	132	132	132	132
Sucrose	38.486	38.486	38.486	38.486	38.486
t-Butylhydroquinone	0.014	0.014	0.014	0.014	0.014
Mineral Mix	35	35	35	35	35
Vitamin Mix	10	10	10	10	10
Choline Bitartrate	2.5	2.5	2.5	2.5	2.5
L-Cystine	3	3	3	3	3
Cholesterol	10	10	10	10	10
Bile salt	2	2	2	2	2
Lard	50	50	50	50	50

The diets were prepared according to the AIN-93G formula with some modifications as described in the section of Materials and methods. Control, C; Brewed Bayou No.1, Br1; Boiled Bayou No.1, Bo1; Brewed Bayou No.8, Br8; Boiled Bayou No.8, Bo8

This study was carried out according to the P.R. China legislation regarding the use and care of laboratory animals and was approved by the Animal Ethics Committee of Northwest A&F University (Yangling, China).

### Analysis of metabolic parameters in rats

The concentrations of plasma lipids were measured using an Automatic Chemistry Analyzer (7020, Hitachi, Tokyo, Japan) at Biochemical Clinical Laboratory, Yangling Demonstration Zone Hospital, Yangling, China. The liver lipids extracted by tissue lysate from tissue lipids assay kits were chemically measured using the method as the kits described. The concentrations of liver lipids were measured by a Tissue triglyceride assay kit, E1003-2; Tissue total cholesterol assay kit, E1015; and Tissue free cholesterol assay kit, E1016 (Applygen Technologies Co., Ltd, Beijing, China). Liver was made to be paraffin sections, and its structure was observed by H&E staining method. The fecal total lipids contents were measured by Soxhlet method. The cholesterol and fecal bile acids contents were measured using the Tissue total cholesterol assay kit, E1015 (Applygen Technologies Co., Beijing, China) and Rat Bile Acid ELISA Kit (Nanjing Sen Shellfish Gamma Biotechnology Co., Ltd., Nanjing, China), respectively.

### Statistical analysis

The data were expressed as the mean ± standard errors (SE) and analyzed by SPSS (Version 12.0 for Windows, SPSS Inc., Chicago, IL, USA) using Tukey-Kramer’s multiple comparison post hoc test and *t*-test. Statistical significance was defined as *P* < 0.05.

## Results

### Growth parameters of rats

The growth parameters of rats were not affected by feeding different oatmeals. The initial body weight, final body weight, body weight gain, food intake, water intake and liver weight did not differ from each other significantly among the five groups (Table 2).

**Table 2:** Effects of eating methods on the growth parameters of Sprague–Dawley rats fed with a hypercholesterolemic diet^a^

	C	Br1	Bo1	Br8	Bo8
Intake (g/day)					
Food	25.1 ± 0.4	25.2 ± 0.5	25.3 ± 0.4	25.0 ± 0.3	25.4 ± 0.3
Water	19.8 ± 0.4	18.9 ± 0.5	19.0 ± 0.5	19.0 ± 0.5	19.8 ± 0.4
Weight (g)					
Initial body weight	281 ± 5	279 ± 6	277 ± 4	283 ± 5	279 ± 7
Final body weight	327 ± 9	333 ± 10	332 ± 9	323 ± 13	323 ± 10
Body weight gain	56 ± 9	54 ± 8	55 ± 5	50 ± 7	54 ± 8
Liver	10.7 ± 0.3	10.9 ± 0.8	11.0 ± 0.6	10.9 ± 0.5	11.3 ± 0.2

^a^Means and standard errors (SE) were determined from 8 rats per group. Control, C; Brewed Bayou No.1, Br1; Boiled Bayou No.1, Bo1; Brewed Bayou No.8, Br8; Boiled Bayou No.8, Bo8

### Plasma lipids

**Fig. 1:**
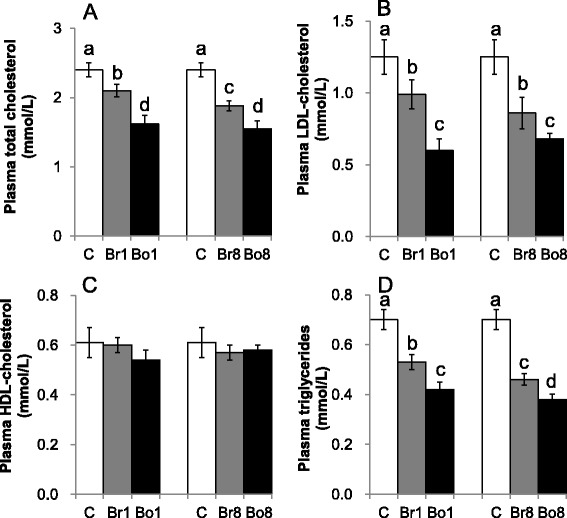
Plasma total cholesterol (**a**), LDL-cholesterol (**b**), HDL-cholesterol (**c**), and triglycerides (**d**) of the experimental rats. Means and standard errors were determined from 8 rats per group. Different superscript letters indicate significant differences at *P* < 0.05

Both boiled and brewed oatmeal prevented the diet-induced increase of the concentrations of plasma total cholesterol, LDL-cholesterol and triglycerides as compared to the control group (*P* < 0.05) (Fig. 1a, b, d). The capabilities of boiled oatmeal, both Bo1 and Bo8, in lowering plasma total cholesterol, LDL-cholesterol and triglycerides concentrations were more significant than those of brewed oatmeal (*P* < 0.05) (Fig. 1a, b, d). Moreover, the Br8 prevented the diet-induced increase of the plasma total cholesterol concentration more significantly than the Br1 (*P* < 0.05) (Fig. 1a). The plasma triglycerides concentrations in both Br8 and Bo8 were significantly lower than those in both Br1 and Bo1 (*P* < 0.05) (Fig. 1d). There was no difference in the concentrations of plasma HDL-cholesterol among the five groups (Fig. 1c).

### Liver lipids

**Fig. 2:**
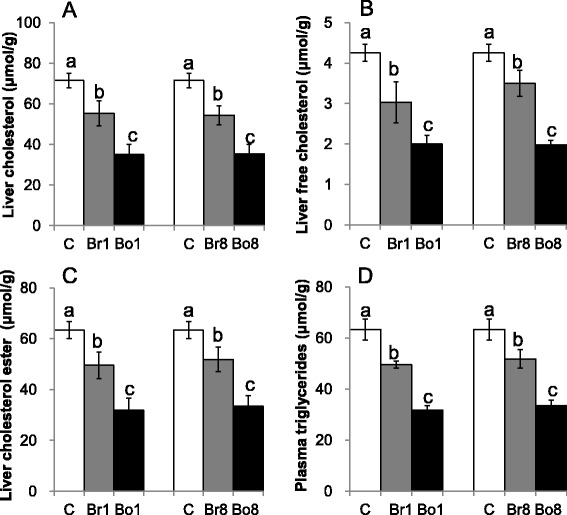
Liver total cholesterol (**a**), free cholesterol (**b**), cholesterol ester (**c**), and triglycerides (**d**) of the experimental rats. Means and standard errors were determined from 9 rats per group. Different superscript letters indicate significant differences at *P* < 0.05

**Fig. 3:**
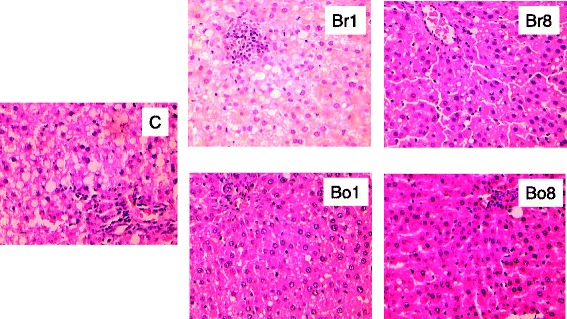
Liver structure of the experimental rats (H&E stain; 200×)

As shown in Fig. 2, the concentrations of liver cholesterol, free cholesterol, cholesterol ester and triglycerides in Brewing Bayou NO.1 (Br1), Boiling Bayou NO.1 (Bo1), Brewing Bayou NO.8 (Br8) and Boiling Bayou NO.8 (Bo8) groups were significantly lower than those in the control group (*P* < 0.05). Moreover, the effect of boiled groups in lowering liver lipid concentrations was stronger than that of brewed groups (*P* < 0.05). H&E stained liver structure was showed in Fig. 3. These liver lipids concentrations were not significantly different between two varieties.

### Fecal lipids

The fecal total lipids excretion, cholesterol and bile acids excretion were increased in oatmeal groups compared with the control group (*P* < 0.05) (Table 3). There was no difference in fecal weight among the 5 groups. The capabilities of boiled oatmeal, both two varieties, to increase fecal total lipids excretion, cholesterol and bile acids were stronger than that of brewed oatmeal (*P* < 0.05). The ability of Br8 to increase bile acids was stronger than that of Br1 (*P* < 0.05). In addition, the ability of Bo8 to increase fecal total lipids excretion, cholesterol and bile acids was stronger than that of Bo1 (*P* < 0.05).

**Table 3:** Effects of eating methods on the fecal parameters of Sprague–Dawley rats fed with a hypercholesterolemic diet^1^

	C	Br1	Bo1	Br8	Bo8
Fecal weight (g/day)	2.50 ± 0.21	2.55 ± 0.23	2.61 ± 0.17	2.58 ± 0.19	2.66 ± 0.11
Fecal lipids (mg/day)					
Total lipids	91 ± 9^a^	132 ± 11^b^	197 ± 10^c^	136 ± 8^b^	241 ± 22^d^
Cholesterol	44 ± 5^a^	59 ± 6^b^	90 ± 8^c^	64 ± 7^b^	117 ± 13^d^
Bile acids	57 ± 3^a^	67 ± 5^b^	93 ± 4^c^	89 ± 5^c^	121 ± 11^d^

^1^Means and standard errors (SE) were determined from 8 rats per group. Different superscript letters indicated significant differences at *P* < 0.05 (Tukey-Kramer’s multiple comparison post hoc test). Control, C; Brewed Bayou No.1, Br1; Boiled Bayou No.1, Bo1; Brewed Bayou No.8, Br8; Boiled Bayou No.8, Bo8

### Chemical and physical characteristics of oatmeal

**Fig. 4:**
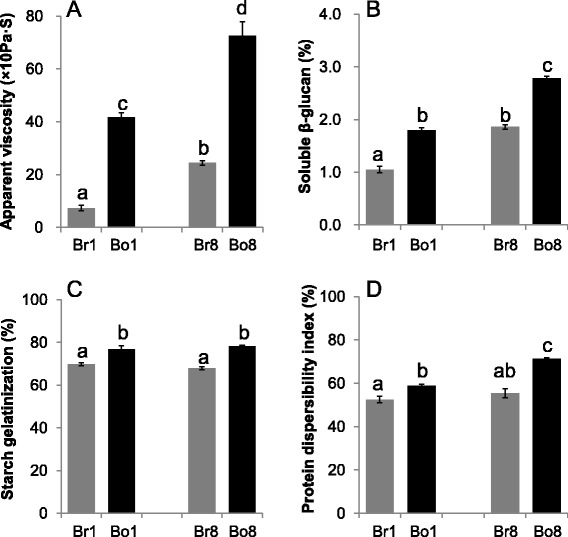
Apparent viscosity (**a**), soluble β-glucan (**b**), starch gelatinization (**c**), and protein dispersibility index (**d**) of the brewed oatmeal and boiled oatmeal. Means and standard errors were determined in triplicate for each sample. Different superscript letters indicate significant differences at *P* < 0.05

The brewing and boiling methods had different effects onthe apparent viscosity, soluble β-glucan concentration, starch gelatinization and PDI of oatmeal (Fig. 4). The boiled oatmeal had higher apparent viscosity, soluble β-glucan content, gelatinization index and PDI (*P* < 0.05) than the brewed one. The apparent viscosity, soluble β-glucan content and PDI of Bo8 and Br8 were significantly higher than those of Bo1 and Br1 (*P* < 0.05) (Fig. 4a, b, d).

## Discussion

Numerous studies have revealed the cholesterol metabolism of oats in animal models and hypercholesterolemic subjects may play the beneficial effects, and thereby reduce the risk of cerebrovascular disease [1, 2, 19, 20]. So far, a large number of studies have focused on the effects of oat functional ingredients and its mechanism, but the effect of cooking method of oatmeal on cholesterol-lowering activity is still unknown. In present study, boiling methods has been demonstrated to be a more efficacy cooking method for oatmeal for improving its effects in lowering plasma lipid concentration. Boiled oats (both two varieties) lowered the concentrations of plasma total cholesterol, LDL-cholesterol and triglycerides more significantly than the brewed oatmeal. In addition, the ability of boiled oatmeal to lower liver lipids concentrations was stronger than that of brewed oatmeal (*P* < 0.05). The findings were new because the effects of cooking methods on oatmeal cholesterol-lowering activity was not reported much elsewhere. We also found that Bayou No.8 had better lowering effect of plasma lipids than Bayou No.1. It was in agreement with the reports that the cholesterol-lowering effect was attributed to its β-glucan content [9, 21].

It has been widely reported that dietary oats reduces the plasma lipids concentrations by promoting the excretion of fecal total lipids and bile acids, and regulating the activities of 3-hydroxy-3-methyl glutaryl-coenzyme A reductase and cholesterol 7-α hydroxylase CYP7A1 [22]. In present study, the fecal total lipids, cholesterol and bile acids excretion were increased in all of oatmeal groups compared with the control group (*P* < 0.05). The capability of boiled oatmeal to increase fecal lipids excretion was stronger than that of brewed oatmeal (*P* < 0.05). The results indicate that the boiling method contributes to decrease plasma and liver cholesterol concentrations by strengthening the inhibition of cholesterol absorption in intestine through high intestinal viscosity provided by high soluble β-glucan content.

Studies have shown that the highly water-soluble β-glucan plays the better role in lowering cholesterol than β-glucan with the low water-solubility [10, 12]. The soluble β-glucan shows the capacity of forming highly viscous solutions which seems to be responsible for the reduction of plasma cholesterol by increasing intestinal viscosity [14–16]. In addition, the soluble β-glucan is fermented by the intestinal microflora to produce the short chain fatty acids (SCFAs) which also results in the decrease of cholesterol [23]. Bell et al. [24] reported that these SCFAs, absorbed through the portal vein, improved cholesterol metabolism by increasing catabolism of LDL-cholesterol or regulating the related enzymes activities. The boiled oatmeal had higher soluble β-glucan content (Fig. 4a) and apparent viscosity (Fig. 4b) than the brewed oatmeal (*P* < 0.05), which might be related to the higher efficacy of boiled oatmeal in lowering lipids. Our study was in agree with Izydorczyk et al. [13] which involved in hull-less barley. The boiled oatmeal showed higher starch gelatinization and PDI compared with the brewed oatmeal (*P* < 0.05) (Fig. 4c, d). Because the boiling applied more thermal energy which results in the increase of starch gelatinization and PDI, boiled oatmeal has a loose organizational structure. This may be the reason why boiling increases soluble β-glucan content of oatmeal, thus showing better lipid-lowering effectiveness than brewing.

## Conclusions

The present study clearly revealed that cooked oatmeal can lower plasma and liver lipids concentrations in Sprague–Dawley rats fed with a hypercholesterolemic diet, and boiled oatmeal is more effective than the brewed one. The boiled oatmeal improved cholesterol metabolism by enhancing the excretions of fecal total lipids, cholesterol and bile acids. Furthermore, the better hypocholesterolemic effect of boiled oatmeal was proven to be positively correlated to its higher soluble β-glucan content and apparent viscosity as compared to the brewed oatmeal.
